# Virus Resistance Is Not Costly in a Marine Alga Evolving under Multiple Environmental Stressors

**DOI:** 10.3390/v9030039

**Published:** 2017-03-08

**Authors:** Sarah E. Heath, Kirsten Knox, Pedro F. Vale, Sinead Collins

**Affiliations:** 1 Institute of Evolutionary Biology, School of Biological Sciences, University of Edinburgh, Ashworth Laboratories, The King’s Buildings, Charlotte Auerbach Road, Edinburgh EH9 3FL, UK; Pedro.Vale@ed.ac.uk (P.F.V.); s.collins@ed.ac.uk (S.C.); 2 Institute of Molecular Plant Sciences, School of Biological Sciences, University of Edinburgh, Rutherford Building, Max Born Crescent, Edinburgh EH9 3BF, UK; kirsten.knox@ed.ac.uk

**Keywords:** evolution, trade-off, cost of resistance, Phycodnavirus, Prasinovirus, environmental change, virus-host interactions, marine viral ecology, *Ostreococcus tauri*

## Abstract

Viruses are important evolutionary drivers of host ecology and evolution. The marine picoplankton *Ostreococcus tauri* has three known resistance types that arise in response to infection with the Phycodnavirus OtV5: susceptible cells (S) that lyse following viral entry and replication; resistant cells (R) that are refractory to viral entry; and resistant producers (RP) that do not all lyse but maintain some viruses within the population. To test for evolutionary costs of maintaining antiviral resistance, we examined whether *O. tauri* populations composed of each resistance type differed in their evolutionary responses to several environmental drivers (lower light, lower salt, lower phosphate and a changing environment) in the absence of viruses for approximately 200 generations. We did not detect a cost of resistance as measured by life-history traits (population growth rate, cell size and cell chlorophyll content) and competitive ability. Specifically, all R and RP populations remained resistant to OtV5 lysis for the entire 200-generation experiment, whereas lysis occurred in all S populations, suggesting that resistance is not costly to maintain even when direct selection for resistance was removed, or that there could be a genetic constraint preventing return to a susceptible resistance type. Following evolution, all S population densities dropped when inoculated with OtV5, but not to zero, indicating that lysis was incomplete, and that some cells may have gained a resistance mutation over the evolution experiment. These findings suggest that maintaining resistance in the absence of viruses was not costly.

## 1. Introduction

Viruses are the most abundant biological entities in the oceans, with an estimated 10^30^ particles globally [1]. Viruses play a key role in marine food webs, partially because viral infection of unicellular organisms often results in cell lysis, where the infected cell bursts to release the new viruses; products of lysis feed back into the microbial loop and provide organic matter to organisms at the base of the food web daily [2]. In addition to being a large cause of mortality to their hosts, viruses can exert strong selection on host immune defense, leading to the evolution of host resistance mechanisms. Strong immune defenses, in turn, impose strong selection on viruses to evade these resistance responses leading to an ongoing co-evolutionary process between hosts and viruses [3]. Experimental evidence of host-virus coevolution has come mainly from bacteria-phage systems [3,4]. Viruses evolve rapidly due to their small size and high mutation rates [5] which can strongly influence the evolution of their hosts. However, in addition to infection, hosts are also subject to other selection pressures, such as severe or stressful environmental changes. In the case of marine hosts, they will be subject to natural selection both from their viruses, and from, for example, the changes in nutrients, temperature and light associated with global change in the oceans [6], which opens up the possibility that the genetic and physiological changes associated with resistance may affect host evolution in response to challenges other than the virus itself. This in turn has the potential to affect how primary productivity at the base of marine food webs evolves in response to global change. Studies have examined environmental effects on interactions between microalgae and their viruses under a range of conditions including changes in temperature [7,8], nutrients [9,10,11,12,13], UV radiation [14], light intensity [11,15,16], and CO_2_ levels [13,17,18]. Environmental change can have direct effects on marine viruses, for example by damaging and/or deactivating the particles through UV exposure or extreme temperatures [8,14]. However, viral abundance is thought to be mainly dependent on host availability and, therefore, the effects of environmental change on viruses are expected to be mainly indirect (e.g., [19]). Here we focus on host evolution rather than viral selection.

Hosts are capable of evolving resistance to their viruses, though resistance often entails a fitness cost, which can vary in form and magnitude [20]. Costs of resistance that have been reported in microorganisms include reduced competitive ability [20,21], reduced growth rate [22,23], reduced original function of a receptor protein [24,25], and increased susceptibility to other viruses [26,27,28]. If the cost of resistance is substantial and related to growth or competitive ability, resistance might be lost when the selection pressure for it is removed (i.e., when viruses are absent) [29]. For example, under conditions where viruses are present and able to interact with their host cells, resistant hosts should have a selective advantage over susceptible hosts by avoiding lysis. However, in the absence of viruses, the selection pressure for resistance is removed and costs of resistance, if present and substantial, should reduce host fitness, so that there is an advantage to losing resistance. Most studies have focused on costs of resistance in bacteria (e.g., [22,28,30,31]), however data for eukaryotic microalgae are lacking, which limits our ability to translate the literature on host-virus interactions to primary producers in the oceans. Because marine algae are the dominant primary producers in oceans [32], changes in the abundance, distribution and composition of microalgal assemblages in response to climate change are likely to have important implications for marine communities.

The marine picoeukaryote *Ostreococcus tauri* and its viruses, *Ostreococcus tauri* viruses (OtVs), are abundant in Mediterranean lagoons [33]. OtVs are lytic viruses belonging to the family Phycodnaviridae that cause susceptible (S) host *O. tauri* cells to burst following infection [34]. However, two resistant host types have been observed [35,36]. In the first type, viruses can attach to the resistant (R) host cells but are unable to replicate and cause lysis. In the second type, resistant producer (RP) populations consist mainly of resistant cells with a minority of susceptible cells (<0.5%) that maintains a population of viruses. These two resistance mechanisms have been observed repeatedly and remain resistant to lysis over many generation of sub-culturing [35,36]. Previous work found that there was no difference in growth rates between the three resistance types when they were maintained separately under standard laboratory culturing conditions, although long term competitions indicated a cost of resistance with susceptible cells outcompeting resistant cells and resistant cells outcompeting resistant producers after 100 and 200 days, respectively [35].

This study examined whether a cost of resistance could be detected in *O. tauri* in terms of the ability to adapt to different environmental conditions, and whether the evolutionary responses to environmental change were affected by resistance type. Populations of S, R and RP *O. tauri* were evolved under different environmental conditions in the absence of viruses for 200 generations to answer whether resistance type was maintained and how resistance type affected evolutionary responses, even in the absence of coevolutionary dynamics imposed by the presence of viruses. We found that all R and RP populations remained resistant to OtV5 inoculation across all environments, whereas S populations had a lower proportion of cell lysis at the end than at the start of the evolution experiment. Additionally, resistance type affected cell division rates, size and chlorophyll content, whereas selection environment affected cell division rates and competitive ability.

## 2. Materials and Methods

### 2.1. Susceptible and Resistant Lines

*O. tauri* lines were obtained from N. Grimsley, Observatoire Océanologique, Banyuls-sur-Mer, France. Three susceptible lines (NG’2, NG’3 and NG’4), three resistant lines (NG5, NG’13 and NG26) and three resistant producer lines (NG’10, NG’16 and NG27) were used. All lines were derived from a single clone of *O. tauri* (RCC4221) and therefore had the same starting genotype.

### 2.2. Culturing Conditions

For each of the nine lines described above, three biological replicates were evolved per environment (27 independent populations in total per environment). We refer to each independent replicate as a population. Populations were grown in batch culture. Culture medium was prepared using 0.22 μm filtered Instant Ocean artificial seawater (salinity 30 ppt) supplemented with Keller and f/2 vitamins [37]. Control cultures were maintained in a 14:10 light:dark cycle at 85 μmol photon m^−2^ s^−1^ at a constant temperature of 18 °C (Table 1). Each population was grown in 20 mL media and each week, 200 μL was transferred to fresh media to ensure populations were always growing exponentially. Cultures were resuspended by gentle shaking every 2–3 days to prevent cells sticking to the bottom of the flask. For the evolution experiment, *O. tauri* populations were grown either in the control environment as described above, in low light, low phosphate, low salinity or high temperature (Table 1), or a changing environment (random) in which one of the environments from those listed was chosen at random at each transfer. We refer to the environments where the populations evolved as “selection environments”. Populations were grown in the absence of viruses for 32 weeks, corresponding to approximately 200 generations.

For the low light environment, culture flasks were wrapped in 0.15 neutral density foil to reduce light intensity. For the low phosphate environment, phosphate was reduced by preparing Keller medium with half the amount of β-glycerophosphate present in the control media. For low salt, Instant Ocean was added to reach a salinity of 25 ppt. Cultures in the high temperature environment were maintained on a heat mat (Exo Terra Heat Wave substrate heat mat, Yorkshire, UK) set at 20 °C. These selection environments were chosen so that the populations responded to them by changing their growth rates relative to the control environment—in batch culture rapid growth is favored by natural selection, so any environment that decreases growth rates should then result in natural selection for traits that will allow cell division rates to recover in that environment. However, the selection environments were not extreme, so that populations were still able to grow at a measurable rate and survive the dilution rate of the experiment. This is in part so that a similar number of generations elapse in all environments over the course of the experiment.

### 2.3. Testing RP Lines for Viral Production

All resistant producer (RP) lines were tested for viral production prior to the start of the experiment. To check whether the three producing lines (NG’10, NG’16 and NG27) were releasing infectious viruses, we used the supernatant to infect susceptible *O. tauri* strain RCC4221. Two milliliters of each population were transferred to an Eppendorf tube and centrifuged at 8000× *g* for 15 min. Four hundred milliliters of the supernatant were removed carefully without drawing up any of the cells from the pellet at the bottom of the tube, and used to inoculate 1 mL of susceptible *O. tauri*. OtV5 was used as a positive control and Keller media was used as a negative control. Eight replicates were performed before the experiment was started. The test was performed every four weeks with three replicates per population. Samples were checked for lysis either by observing by eye whether they were green or clear, or by measuring cell densities using a BD FACSCanto II (BD Biosciences, Oxford, UK) flow cytometer.

In addition to liquid lysis tests, frozen stocks of RP supernatant were made by adding dimethyl sulfoxide (DMSO) (final concentration 10%) and storing at −80 °C. We tested these samples for viruses using the plaque assay technique [34]. A 1.5% agarose suspension was made and 5 mL aliquots were prepared in Falcon tubes and held at 70 °C in a water bath. In a 50 mL Falcon tube, 30 mL exponentially growing *O. tauri* culture, 15 mL Keller media and 5 mL agarose were mixed rapidly but gently by inverting the tube (final agarose concentration 0.15%). The agarose was poured into a 12 cm square petri dish and left to set. Tenfold serial dilutions of the RP supernatant were made in 96-well plates using one row per sample. A Boekel Replicator was used to transfer all of the serial dilutions from one 96-well plate to one square petri dish. The replicator was sterilized between each use using ethanol and a flame. Petri dishes were checked daily for lysis plaques for a maximum of 10 days.

### 2.4. Testing Resistance Type Using OtV5 Inoculation

OtV5 inoculum was prepared prior to the start of the experiment and stored at −80 °C in 10% DMSO (final concentration) and inoculations were performed from the frozen stocks. The experiment did not include a co-evolving virus which allowed us to measure host evolution relative to the ancestral virus. After 32 weeks of evolution, each population was inoculated with a suspension of OtV5 particles to test whether it was susceptible or resistant to viral lysis. Samples were tested by inoculating 1 mL cell culture at a density of 10^5^ with 10 μL OtV5 in 48-well plates with three replicates for each sample. Negative controls that were not inoculated with OtV5 were used as a comparison of cell growth. Cell density was measured using a FACSCanto flow cytometer 3 days after inoculation. Samples were run on 96-well plates by counting the total number of cells in 10 μL with a flow rate of 2.0 μL per second.

Data were analyzed with linear mixed effects models using the statistical packages lme4 [38] and lmerTest [39] in R (version 3.2.0, R Core Team, Vienna, Austria) to identify differences in cell densities after OtV5 inoculation compared to controls that were not inoculated. Selection environment, resistance type and treatment (inoculated or not inoculated) were set as fixed effects with population as a random effect. Post hoc Tukey tests were performed using lsmeans to confirm where significant differences occurred within the different effects.

### 2.5. Population Growth Rates, Cell Size and Cell Chlorophyll Content after Evolution

At the end of the evolution experiment, we quantified evolutionary responses by measuring average cell division rates and by measuring cell size and chlorophyll content for each population. All evolved populations were assayed in their selection environment and in the control environment, and all control populations were assayed in all selection environments except high temperature, since all populations in the high temperature environment went extinct and therefore there were no high temperature evolved strains. The populations that had evolved in a random environment for each transfer were only assayed in the control environment, which was not one of the environments they had been exposed to during the experiment, meaning only a correlated response (rather than a direct response) to selection could be obtained. Each population was assayed in triplicate. Due to the size of the experiment, assays were divided randomly into seven time blocks. This was factored into the statistical analysis.

Average cell division rates, which we refer to as “growth rates” are the average number of cell divisions per day over seven days, which corresponds to one transfer cycle. All populations were first maintained in their assay environment for an acclimation period of one week, which was one full transfer cycle, prior to measuring growth rates. After acclimation, cells were counted using a FACSCanto flow cytometer before the transfer into the assay environment (to calculate the number of cells transferred into fresh media) and again after seven days of growth. Each sample was counted in triplicate. The cell counts were converted to cells per milliliter and the number of divisions per day was calculated using Equation (1).

(1) $$\mu \;\left(d^{-1}\right)=\frac{\log_{2}\;\left(\frac{N_{t}}{N_{0}}\right)}{t-t_{0}}$$

where μ is population growth rate, and N_t_ and N_0_ are the cell densities (cells mL^−1^) at times t and t_0_ (days), respectively. This measures the average number of cell divisions per ancestor over a single growth cycle and allows a comparison of offspring production between environments even if there are differences in the shape of the population growth curve, or in cases where r cannot be accurately estimated. To avoid biases of cell divisions being dependent on the time of the cell cycle, cells were always measured at the same time of day (at the beginning of the light period when cells are in G1 phase).

Cell size was inferred from FSC (forward scatter), which was calibrated using beads of known sizes (1 μm, 3 μm and 6.6 μm). Chlorophyll fluorescence was inferred by measuring PerCP-Cy5.5 emission with excitation at 488 nm. Relative chlorophyll was analyzed by taking the average chlorophyll fluorescence for all susceptible strains in the control environment and setting this to a value of 1, with chlorophyll measurements of all other strains relative to this value.

Data were analyzed with linear mixed effects models. To analyze differences in growth rate, cell size and chlorophyll under different environments, selection environment, assay environment and resistance type were fixed effects and population and block ware random effects that were treated as un-nested. An additional model was fitted to examine whether there was a difference in growth rate when populations were assayed in their selection environment or when they were assayed in a different environment, with assay as the only fixed effect and population and block set as random effects.

### 2.6. Competition Assay

To measure competitive fitness, all evolved populations were competed against a green fluorescent protein (GFP) line of *O. tauri*. A Gateway enabled entry clone containing roGFP2 was obtained by linearizing pH2GW7-roGFP2 [40] with EcoRV. The linearized vector was recombined with pDONR207, creating a pDONR207-roGFP2 clone. A pOtOX binary vector [41] was adapted to become a Gateway^®^ destination vector and pDON207-roGFP2 was recombined into the vector, downstream of the high-affinity phosphate transporter (HAPT) promoter [41]. The pOtOx-roGFP2 vector was subsequently transformed into *O. tauri* using the procedure previously described [42].

All evolved populations competed in the selection environment that they evolved in, and all control populations competed in the control environment as well as in each selection environment to measure plastic response. All of the random populations competed in the control environment. All populations, including the roGFP line, were acclimated for one week in the corresponding assay environment prior to the assay. Equal starting densities of 5 × 10^5^ of each evolved population and the roGFP line were grown in 20 mL media for one week, after which cells were counted using a FACSCanto flow cytometer. GFP and non-GFP populations were distinguished by measuring fluorescein isothiocyanate A (FITC-A) emission at 519 nm with excitation at 495 nm. Competitiveness of the evolved populations was measured relative to the roGFP line as fold change in cell density. Data were analyzed with a linear mixed effects model, with selection environment, assay environment and resistance type as fixed effects and population and assay replicate as random effects.

## 3. Results

### 3.1. Susceptibility to OtV5 after Evolution

#### 3.1.1. Host Resistance Type Was Maintained during Evolution

**Figure 1:**
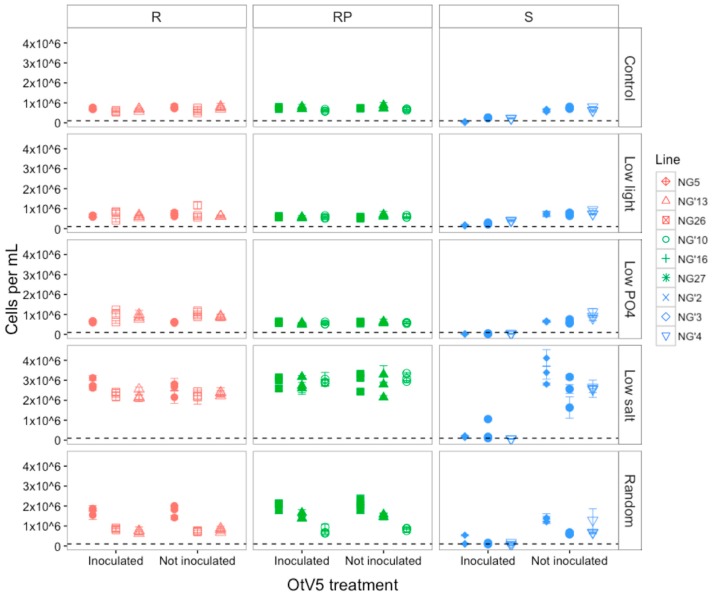
Mean (± SE) cell density mL^−1^ of resistant (R), resistant producer (RP) and susceptible (S) *O. tauri* lines three days after OtV5 inoculation in five environments. Points represent the average of the three assay replicates for each evolved population. Inoculated = populations inoculated with OtV5, Not inoculated = negative control populations that were grown for the same period without OtV5 inoculation. There were three evolved populations of each line. The dashed line represents the starting cell density at 100,000 cell mL^−1^.

After 200 generations of evolution in the selection environments, all surviving R and RP populations remained resistant to OtV5 lysis and all S populations remained susceptible to viral lysis in those environments (Figure 1). A significant interaction between selection environment, resistance type and treatment (OtV5 inoculation) affected susceptibility of *O. tauri* to OtV5 (ANOVA environment × resistance type × treatment, *F*_8,238_ = 15.22, *p* < 0.0001). A post hoc Tukey test showed that this was due to cell lysis of S populations (t_8,238_ = 10.66, *p* < 0.001), whereas cell density of R and RP lines did not decrease compared to controls that were not inoculated. The highest cell densities were observed in the low salt (post hoc Tukey test, t_8,238_ = −29.90, *p* < 0.0001) and random (post hoc Tukey test, t_8,238_ = −7.54, *p* < 0.0001) environments. The OtV5-inoculated S populations in low phosphate were the only populations where cell density fell below the starting cell density across all populations, indicating almost complete cell lysis and no cell growth for this combination of resistance type and selection environment. R and RP lines did not show decreases in cell density after inoculation with OtV5 compared to controls that were not inoculated, whereas S lines did.

R and RP populations did not show a significant difference in cell density between populations that had been inoculated with OtV5 and populations that had not (Figure S1). In contrast, all S populations inoculated with OtV5 showed a change in cell density relative to non-inoculated S populations in the same environments (ANOVA effect of resistance type on difference *F*_2,125_ = 66.51, *p* < 0.0001). The largest differences in cell densities between inoculated and non-inoculated populations were observed in S populations evolved in the low salt environment, showing that whilst all populations in this environment were able to reach high densities in the absence of viruses, they were unable to grow in the presence of OtV5 (Figure 1). The large difference in S populations in low salt was due to the high growth rate of populations that had not been inoculated, since inoculated populations did not fall to lower densities than inoculated S populations in any other environments.

#### 3.1.2. OtV5-Mediated Lysis Decreased in Susceptible Populations

**Figure 2:**
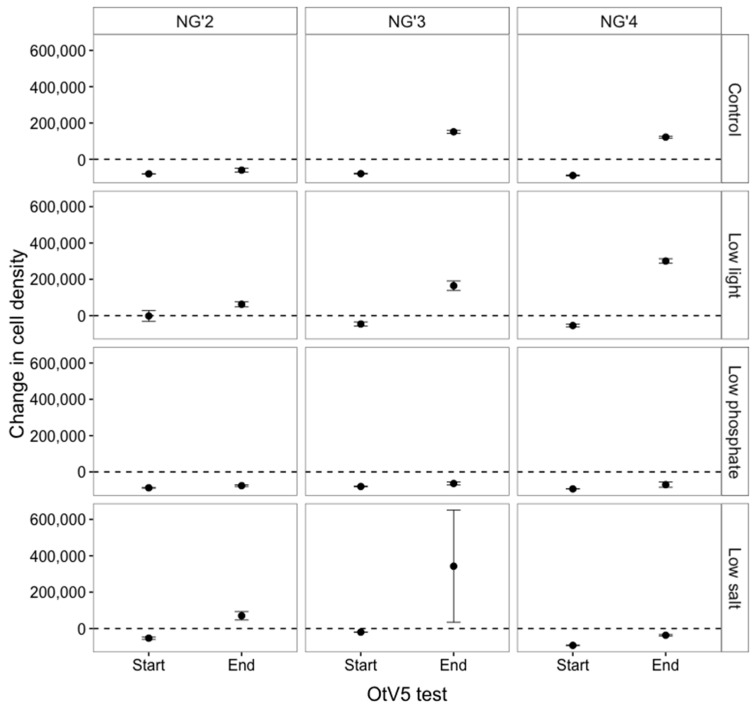
Change in cell density of the susceptible lines NG’2, NG’3 and NG’4 after OtV5 inoculation one week into the selection experiment (Start) and after 32 transfer cycles of evolution (End). The dashed line represents no change.

Although S populations remained sensitive to viral lysis at the end of the evolution experiment, complete lysis was not observed in all populations, with a small proportion of populations able to reach numbers above the starting density of 100,000 cells mL^−1^ (Figure 1). This was in contrast to the beginning of the evolution experiment, when all susceptible populations fell below 100,000 cells mL^−1^ after inoculation with OtV5, indicating near-complete lysis (ANOVA effect of time point on cell density, *F*_1,65_ = 21.87, *p* < 0.0001) (Figure 2). The highest proportion of S cells that did not lyse was found in low salt evolved populations, suggesting that resistance mutations had been maintained in this environment, despite no selection by OtV5. To eliminate the possibility that the infection dynamics had changed and that the population decline was still in process, we measured the population density seven days after inoculation and did not observe any further decrease in population density (Figure S2).

#### 3.1.3. RPs Stopped Producing Viruses Early in the Evolution Experiment

During the evolution experiment, RP populations (NG27, NG’10 and NG’16) were tested to check that they were still producing viruses. Seven transfer cycles into the evolution experiment, all NG27 populations in all environments were still producing infectious viruses, as observed by cell lysis when their supernatant was used to inoculate the susceptible *O. tauri* strain RCC4221. In contrast, RCC4221 cultures that were inoculated with the supernatant of all populations of NG’10 and NG’16 continued growing, showing that no observable lysis had occurred. After 17 transfers in the selection environments, all RP populations in all environments had stopped producing infectious viruses (Figure S3), as observed by flow cytometric cell counts of RCC4221 populations inoculated with the supernatant of RP populations. When it was clear that all RP populations had stopped producing infectious viruses, frozen supernatant samples collected at transfers 9, 12, 14 and 15 were tested using the plaque assay method. No plaques were observed in any samples tested, thus we concluded that all RP populations in all environments had stopped producing viruses within nine weeks of the selection experiment.

### 3.2. Changes in Trait Values after Evolution

#### 3.2.1. Changes in Cell Division Rate and Population Persistence during the Selection Experiment

**Figure 3:**
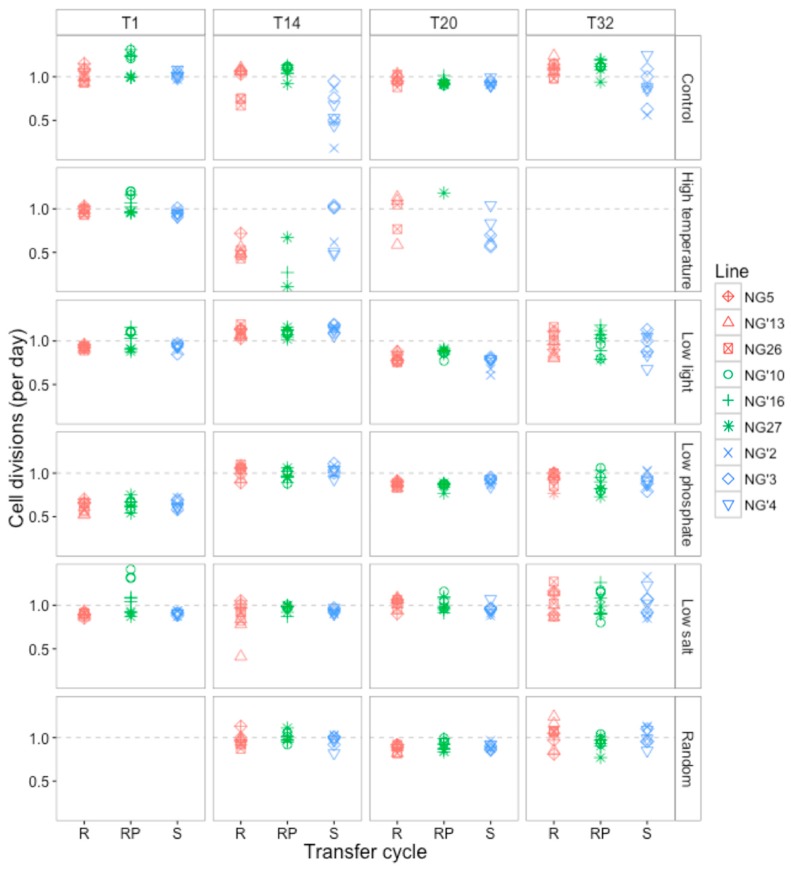
Growth rates as measured by mean cell divisions per day for each evolving population over four time points (1, 14, 20 and 32 transfer cycles). The dashed line represents one cell division per day. T1 is the growth rate following acclimation at the beginning of the experiment. There are no growth measurements for the randomized environment at T1 because lines had only been growing for one transfer cycle.

Here, we focus on how growth rates vary with resistance type, selection environment and the number of transfer cycles in the selection environment. Growth rates of all populations were measured as the number of cell divisions per day, at four time points during the experiment (including at the beginning and end) (Figure 3). When comparing these time points, growth was significantly affected by environment, resistance type and time point (*p* < 0.0001 for all effects). In the first transfer cycle, which measured the population growth rates at the very start of the experiment following one week of acclimation, two out of the three RP lines (NG’10 and NG’16) had increased growth rates across all environments except for low phosphate (ANOVA effect of growth rate on cell divisions, *F*_3,5_ = 17.19, *p* = 0.046). These results are reported in [43].

After 14 transfer cycles, growth rates of all populations were approximately one division per day in the high salt, low phosphate, low light and random environments (Figure 3). In the control environment, growth rate varied across all S lines, even between populations of the same starting line, ranging from 0.18 to 0.87 divisions per day. The increased growth of all lines evolving in low phosphate to one division per day, which is the normal growth rate reported for *O. tauri* in phosphate-replete media, is consistent with adaptation to low phosphate in less than 100 generations. Additionally, RP lines that had been dividing more rapidly at transfer 1 were dividing at the same rate as other lines within each environment (Figure 3). This may be because the RP populations had stopped producing viruses and shifted to the R resistance type (see Section 3.1.3), thereby losing the growth advantage associated with the RP resistance type early on in this experiment. By transfer 24, all populations in the high temperature environment had gone extinct. RP populations went extinct more quickly than S and R populations, with 66% of RP lines extinct by T14 compared to 33% and 22% of S and R, respectively (Figure 3). At transfer 20, only three high temperature populations remained: one S (NG’4) and two R (NG’13 and NG26).

#### 3.2.2. Growth Rates Varied with Selection Environment and Assay Environment after Evolution

After approximately 200 generations of evolution in each environment, a transplant assay was performed to quantify environmental effects on population growth rate, cell size and cell chlorophyll content for each evolved population. Here we define the selection environment as the environment that the population evolved in, and the assay environment as the environment in which measurements were taken. The direct response to selection compares the growth rate of a population evolved in a given selection environment with the growth rate of a population evolved in the control environment when both are grown (separately) in that given selection environment. The effect of selection environment on the direct response to evolution was large, and driven by the direct response to selection in the low phosphate environment (ANOVA effect selection environment on direct response, *F*_2,228_ = 9.26, *p* = 0.0001), whereas the effect of resistance type was smaller (ANOVA effect of resistance type on direct response, *F*_2,228_ 2.87, *p* = 0.06).

**Figure 4:**
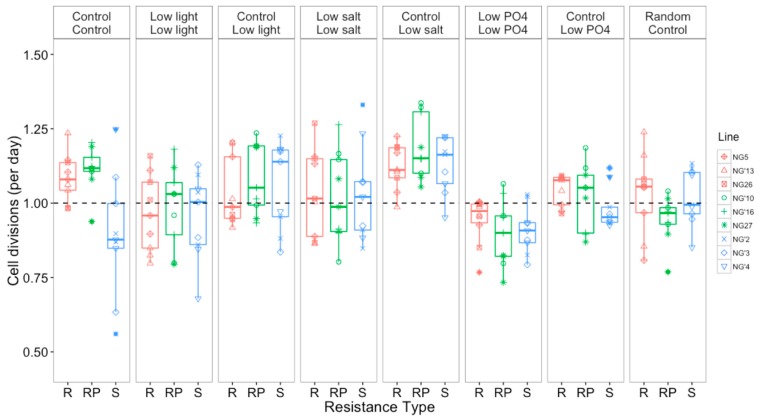
Mean cell divisions per day (±SEM). R = resistant, RP = resistant producer, S = susceptible. Each panel represents a growth assay, with cells evolved in the selection environment (top label) and growth rates measured in the assay environment (bottom label). The dashed line indicates, for reference, one cell division per day.

Selection environment alone and assay environment alone both had a significant effect on population growth rate (ANOVA effect of selection environment on growth, *F*_4,200_ = 19.92, *p* < 0.0001; ANOVA effect of assay environment on growth, *F*_3,758_ = 32.43, *p* < 0.0001), which shows that environment affected growth rates. Resistance type also had an effect on growth rate (ANOVA effect of resistance type on growth, *F*_2,195_ = 4.21, *p* = 0.02), with R populations having the fastest cell division rates and S populations having the slowest cell division rates. Additionally, an interaction between selection environment and assay environment affected growth rate, indicating that the way in which selection environment affected growth differed between assay environments (ANOVA selection environment × assay environment, *F*_3,757_ = 2.89, *p* = 0.03). The fastest growth rates were seen in the evolved control populations that were assayed in low salt (Figure 4). Better performance was not due to being assayed in the same selection environment that the populations had evolved in (ANOVA effect of being assayed in selection environment on growth, *F*_1,831_ = 1.70, *p* = 0.19).

#### 3.2.3. Resistance Type Affected Cell Size and Chlorophyll Content

Cells from different resistance types had different cell sizes (ANOVA effect of resistance type on size, *F*_2,140_ = 9.49, *p* = 0.0001) (Figure S4) and this was not affected during evolution in any of the environments (ANOVA effect of selection environment on size, *F*_4,155_ = 0.66, *p* = 0.62; ANOVA effect of assay environment on size, *F*_3,735_ = 1.60, *p* = 0.19). The greatest variation in cell size between populations was observed when control-evolved cells were assayed in low salt (0.86–0.97 μm) across all resistance types. Less variation was found in the control-evolved cells assayed in low phosphate (0.82–0.97 μm).

The environment in which populations were assayed had a significant effect on the relative chlorophyll content per cell volume (ANOVA effect of assay environment on chlorophyll, *F*_3,744_ = 17.83, *p* < 0.0001). However, selection environment did not (ANOVA effect of selection environment on chlorophyll, *F*_4,168_ = 0.90, *p* = 0.47). Resistance type affected chlorophyll content (ANOVA effect of resistance type on chlorophyll, *F*_2,153_ = 8.54, *p* < 0.0001). Susceptible populations that had been evolving in the control environment contained high amounts of chlorophyll relative to their cell size when assayed under all three selection environments (low light, low salt and low phosphate) (Figure S5).

### 3.3. Selection and Assay Environments Affect Competitive Ability of *O. tauri*

**Figure 5:**
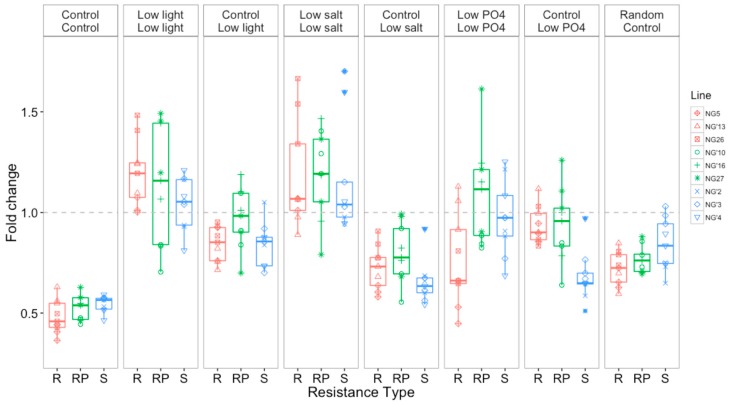
Competitive ability, as measured by fold difference in growth relative to a roGFP-modified *O. tauri* line, of evolved populations and control populations assayed in the selection environments. R = resistant, RP= resistant producer, S = susceptible. Each panel represents one assay, with populations evolved in the selection environment (top label) and competitiveness measured in the assay environment (bottom label). The dashed line represents no change (i.e., equal proportions of roGFP and competitor populations).

In addition to measuring growth rate, size and chlorophyll content, we also tested if costs of resistance could be observed during pairwise competition between each population of S, R, and RP. We measured relative competitive ability, by competing each population against a common competitor harboring a GFP reporter, which allowed us to distinguish between the evolved population and the GFP line. Both selection environment and assay environment affected competitive ability against a roGFP-labeled strain (ANOVA effect of selection environment on competitiveness, F_4,622_ = 16.41, *p* = < 0.0001; ANOVA effect of assay environment on competitiveness, F_3,622_ = 10.96, *p* < 0.0001). Most populations were poor competitors relative to the roGFP line (Figure 5). Lines evolved in low light and low salt were the best competitors. Lines that were assayed in the same environment that they had evolved in were better competitors than control lines that were assayed in the selection environments. This shows that these lines adapted to their selection environment and that growth rate is not necessarily the most appropriate measure of adaptation in this study, which is consistent with other studies in *Ostreococcus* spp. [44]. Interestingly, populations in the control environment were the worst competitors, regardless of resistance type, with a 0.56 mean fold change, showing that all populations were out-competed by the roGFP line. This indicates that the control environment did in fact exert less selection on the populations than did the other environments.

Resistance type alone did not affect competitive ability (ANOVA effect of resistance type of competitiveness, F_2,622_ = 1.22, *p* = 0.30). Although competitive ability differed between resistance types, the response was not consistent across assay environments, with no one resistance type consistently being a better or poorer competitor.

## 4. Discussion

We examined whether cost of resistance varied with the abiotic environment in which *O. tauri* populations evolved. A cost of resistance can manifest in different ways depending on the interaction between host and virus and on the way in which resistance is acquired (e.g., entry of the virus into the cell, and ability of the virus to replicate within the cell and cause lysis). This means that it is often difficult to detect a cost of resistance, so we measured three host responses: ability to maintain resistance, population growth rate and competitive ability.

### 4.1. Susceptibility to OtV5 Did Not Change after Evolution

After evolution in a new environment, OtV5 was still able to lyse susceptible (S) *O. tauri* populations under all environmental conditions tested, whereas R and RP populations remained resistant under all environments, despite the absence of selection pressure for viral resistance (Figure 1). Resistance to pathogens often comes at a fitness cost, such that a proportion of susceptible individuals remain in the population, thereby allowing viruses to persist [21]. If resistance does carry a fitness cost, populations should revert to susceptibility over time, in the prolonged absence of viruses, even if that cost is low, because susceptible cells have a fitness advantage in the absence of viruses [29]. Our study indicated that if there is a cost to simply maintaining resistance in *O. tauri*, it is small. Over the time scale of our experiment, the fitness advantage of susceptible types in the absence of viruses would have to be about 0.005 for a mutation conferring susceptibility in a resistant background to be fixed in the population following a spontaneous reversion of a resistant cell (where we calculate s from ½s/(1−e^−2sN^), and assume a starting frequency of 1/N [45]).

It is possible that there is a genetic constraint preventing the loss of resistance, making the transition from resistant to susceptible phenotypes rare even if resistance is costly. This is consistent with recent studies showing that the resistance mechanism in *O. tauri* is an intracellular response [35] and probably also involves rearrangements of chromosome 19 [36]. The presence of a genetic constraint on losing resistance would favor compensatory mutations that lead to alleles being selected that reduce the cost of resistance [46,47]. Studies evolving *E. coli* in the absence of bacteriophage observed that the cost of resistance to the T4 bacteriophage decreased after 400 generations due to compensatory adaptations [46]. A second possibility is that the cost of resistance to one strain of OtV means increased susceptibility to other virus strains. For example, cyanobacteria can rapidly evolve viral resistance when coevolving with viruses, however increased resistance to one virus can lead to a narrower resistance range thereby making cells more susceptible to other virus strains [27,28]. *O. tauri*-virus interactions can be complex with some OtVs being very specific to host *O. tauri* strains while others are generalists that can infect many strains [26,48]. Our experiment focused only on OtV5 and did not examine evolution of host resistance range.

At the end of the evolution experiment, OtV5 lysed susceptible (S) populations in all environments, but the extent of lysis differed between environments (Figure 1). This could be because one or more resistance mutations had appeared and risen to a detectable frequency in some populations. It is unclear whether incomplete lysis was due to some resistant cells evolving in the susceptible populations, or whether susceptible populations had evolved to make virus entry harder but still possible. Inoculations were performed from frozen stocks, thus OtV5 was not coevolving with the host, enabling us to measure evolution in the *O. tauri* populations relative to the ancestral virus population. We cannot rule out the possibility that there was a slow loss of infective virus titer in the cryopreserved stock, leading to fewer infectious viruses in the inoculum and therefore a lower multiplicity of infection. Physiological changes in susceptible populations arising as an adaptive response to abiotic environmental change did not prevent viral lysis, indicating that viral adsorption was not completely inhibited. This was even evident in the control populations, suggesting that although these populations did not experience a change in environment, they may have evolved changes in cell surface proteins, since were still evolving for the full length of the experiment. However, the biotic environment plays a larger role in resistance acquisition, since resistance to viruses is selected for by the virus [49]. Chemostat experiments to monitor population dynamics in *Chlorella* and *Paramecium bursaria Chlorella* Virus 1 (PBCV-1) showed that control populations maintained in the absence of viruses did not evolve resistance to the ancestor virus, suggesting that resistance arises from host-virus interactions [23]. In contrast, sensitive *E. coli* cells evolved complete resistance to λ infection and resistant cells increased susceptibility to T6* infection after 45,000 generations in the absence of phage [29]. In our experiment, low phosphate was the only environment in which the cell numbers of all lines fell below the starting cell density (Figure 1), suggesting that this environment either affected the infectivity of OtV5 directly or the cells’ response to infection. Other studies report the opposite, with reduced virus infection of algae under low phosphate, possibly due to the requirement of phosphate for viral replication [9,10,13]. Though phosphate levels were low in our experiment, they were sufficient for population growth to be positive, and were higher than found in the Mediterranean Sea [50]. Conflicting results highlight the complexity of host-virus interactions in different study systems as well as different growth conditions.

There was a selection pressure against viral production on RP lines, but not on host resistance across all RP lines in all environments. Similarly, Yau et al. reported that over a two year period RP populations maintained under standard laboratory conditions stopped producing viruses [36]. If RP populations are indeed made up of a majority of resistant cells with a small proportion of susceptible cells arising that lyse upon OtV5 infection, thus maintaining the production of viruses in the media, then we would expect resistance to be selected for in the presence of viruses. Resistance in *O. tauri* is expected to be caused by over-expression of glycosyltransferase genes on chromosome 19 [36]. In this study, the selection environment did not affect the time it took for a selective sweep of resistance to occur in the RP lines, supporting the conclusion that there was little or no selection against resistance, that there is a genetic constraint on losing resistance, or that compensatory mutations enabled resistance to be maintained.

### 4.2. Resistance Type and Environment Affect Evolutionary Response of *O. tauri* to Environmental Change

We did not observe a growth cost of *O. tauri* being resistant to viral lysis, since R populations had the fastest growth overall whereas S populations had the slowest growth. Data on the growth effects of resistance in marine algae are rare. A 20% reduction in growth was reported in the ubiquitous cyanobacterium *Synechococcus* [22], however it is unknown whether viral resistance generally carries a growth cost in eukaryotic algae. Even with no or minimal costs of resistance, the chromosomal rearrangement associated with resistance in *O. tauri* means that the different resistance types have different genetic backgrounds. Therefore, evolution could take different trajectories in hosts with different resistance types due to epistatic interactions between resistance and adaptive changes. For example, trade-off shape varied in response to environmental change and physiological changes of bacteriophage resistant *E. coli*, leading to variation in sensitivity to environmental change across different strains [51]. In our study, when considering the direct response to evolution (which compares the growth rate of the evolved population in its selection environment with the plastic response of the control line in that selection environment), resistance type did not drive direct response. This indicates that the growth response of the three resistance types was similar within environments. If there is an effect of genetic background being introduced by resistance, it is not evident at the level of growth rate under these conditions.

Selection environment affected population growth, with populations evolved in the control environment having the highest growth rates in all assay environments (Figure 4). The decrease in growth in response to our selection environments is consistent with them being of lower quality than the control environment, by design, so that selection was stronger in the non-control environments. Variation in the direct response to evolution was explained by selection environment. Populations evolved in low phosphate had the lowest growth, which is expected when cells are nutrient limited. Interestingly, populations that had evolved in the control environment grew more rapidly in low phosphate than populations that had evolved in low phosphate. This may be because populations that had been evolving in the control environment had enough phosphate reserves within the cell to grow normally for a short period, since growth was only assayed for seven days. Overall, growth rates of populations evolved in the control environment were greater when assayed in the selection environments than the populations that had evolved in those environments, showing that increased growth could be initiated as a stress response, and that cells in the control environment (which was nutrient-replete, and at the optimal temperature and usual salinity for these lines of *O. tauri*) were in better condition overall. The extent of a cost of resistance can be highly dependent on environment. For example, cost of resistance differs when fitness of *E. coli* is measured under different nutrient resources and concentrations [20,52]. We show here that growth rate measurements may not be sensitive enough to detect very small differences between populations conferring a cost of resistance in *O. tauri*, as has also been observed in short term experiments using a single [35] and multiple environments [43]. Studies in bacteria also found that resistant strains grew at the same rate as susceptible strains [21,46]. Our results indicate that, regardless of resistance type, *O. tauri* is able to adapt to environmental change including low light, low salt and low phosphate. However, all populations in the high temperature environment went extinct, despite the modest (2 °C) increase, suggesting that although *O. tauri* can tolerate and grow at higher temperatures over the short-term, sustained temperature increases may exert stronger selection than predicted from short-term studies. It is not possible to infer as of yet whether resistance affects growth rate in natural habitats or whether a cost of resistance is instead associated with tradeoffs that are not related to the abiotic environment, such as resistance to other viral strains.

In contrast to cell division rates, resistance type affected cell size and chlorophyll content, but selection environment did not. Cells in RP populations were sometimes larger in size and S populations were slightly smaller. Often, small size is associated with a response to nutrient limitation, increased temperature and light limitation in phytoplankton [53,54,55,56,57], however all lines in this study showed slightly increased cell size in low phosphate. An increased cell volume has been observed in coccolithophores in response to phosphate limitation suggesting the adaptive strategy is to reduce phosphorous requirements rather than increasing surface area to volume ratio [58].

RP populations had less chlorophyll in most environments, however overall there was substantial variation in chlorophyll content, especially in S populations. When assayed in the control environment, populations that had evolved in low light, low salt, low phosphate and the random environment had lower chlorophyll than did control populations assayed in these same environments. The response of populations evolved in the control environment increasing their relative chlorophyll content when assayed in low light is consistent with responses to light limitation in other green algae [59,60,61]. Here, we show that response of chlorophyll content to environmental change is variable, both with environment and with resistance type. Previous studies in marine microalgae have reported lower reduced chlorophyll content under nutrient limitation [62] and higher chlorophyll content under some optimal salinities [63,64].

### 4.3. Resistance Type Did Not Affect Competitive Ability Regardless of Environment

Reduced competitive ability is often one of the main restrictions for resistance spreading through a population, however resistance type did not affect the competitive ability of evolved populations in our experiment. We found that environment did affect competitive ability, and similarly in bacteria, the environment that populations evolve in, such as the limiting sugar source or spatial heterogeneity, can affect competitive ability, both with and without coevolving phage [51,52,65]. Other studies have reported a trade-off between competitive ability and resistance, whereas here we found no evidence for reduced resistance with increased competitive ability. The nature of a cost of resistance will depend on the genetic or physiological changes to the cell. For example, *E. coli* mutants showed high variability in competitiveness which was associated with resistance strategy, with cross-resistance to phage T7 significantly decreasing competitive fitness by approximately 3-fold [21]. In contrast, competitions with cyanobacteria showed that total resistance (the total number of viruses to which a host strain was resistant) did not affect competitive ability [22]. These examples reveal that the magnitude of the reduced competitiveness trade-off can depend on the specific resistance strategy.

Evolved populations in the non-control environments were better competitors than control populations that had been exposed to the selection environments for the first time (plastic response), indicating that all lines had adapted to their selection environment. Thus, growth rate is not the most appropriate measure of adaptation in *O. tauri*, since the plastic response was to increase population growth rates, and the evolutionary response was to reverse this plastic increase in growth rates, and this strategy was associated with an increase in competitive fitness. Similar results have been reported previously in *Ostreococcus* spp. where populations with high growth rates in monoculture were poorer competitors than those with lower growth rates in monoculture [44].

## 5. Conclusions

Here, we show that there was no detectable cost of resistance to OtV5 as measured by growth rate or competitive ability for *O. tauri* evolved in several different environments, and that resistance to viruses did not affect adaptation to environmental change. Additionally, we found no reversion of R or RP populations to S as tested by exposure to OtV5, whereas lysis occurred in all S populations. Additionally, all RP lines stopped producing viruses within nine weeks of the experiment. This suggests that a shift from susceptibility to resistance is more common than a shift from resistance to susceptibility, regardless of selection environment, at least for the range of environments used here. Our experiment shows that the conditions under which a cost of resistance may occur or affect adaptation in *O. tauri* are not clear in the laboratory. More work is needed to understand the factors that affect host–virus interactions in the marine environment to better understand evolutionary and ecological responses of marine eukaryotic microalgae to environment change.

## Figures and Tables

**Table 1:** A comparison of the control environment and the treatments used for each selection environment used in this study.

Selection Environment	Control	Treatment
Light (μmol m^−2^ s^−1^)	85	60
Phosphate (μM)	10	5
Salinity (ppt)	30	25
Temperature (°C)	18	20

## Supplementary Materials

Supplementary Information is available online at www.mdpi.com/1999-4915/9/3/39/s1. The pHAPT-roGFP line (CCAP 157/4) and its untransformed parent strain (CCAP 157/2) will be publicly distributed by CCAP https://www.ccap.ac.uk/.

## Data

All data is available from DataDryad doi:10.5061/dryad.vr3hk.

## References

[1] Suttle C.A. (2007). Marine viruses—Major players in the global ecosystem. Nat. Rev. Microbiol..

[2] Wilhelm S.W., Suttle C.A. (1999). Viruses and nutrient cycles in the sea. Bioscience.

[3] Koskella B., Brockhurst M.A. (2014). Bacteria-phage coevolution as a driver of ecological and evolutionary processes in microbial communities. FEMS Microbiol. Rev..

[4] Dennehy J.J. (2012). What Can Phages Tell Us about Host-Pathogen Coevolution?. Int. J. Evol. Biol..

[5] Flint S.J., Enquist L.W., Krug L., Racaniella V., Skalka A. (2000). Principles of Virology: Molecular Biology, Pathogenesis, Virus Ecology.

[6] Doney S.C., Ruckelshaus M., Emmett Duffy J., Barry J.P., Chan F., English C.A., Galindo H.M., Grebmeier J.M., Hollowed A.B., Knowlton N. (2012). Climate Change Impacts on Marine Ecosystems. Ann. Rev. Mar. Sci..

[7] Nagasaki K., Yamaguchi M. (1998). Effect of temperature on the algicidal activity and the stability of HaV (Heterosigma akashiwo virus). Aquat. Microb. Ecol..

[8] Wells L.E., Deming J.W. (2006). Effects of temperature, salinity and clay particles on inactivation and decay of cold-active marine Bacteriophage 9A. Aquat. Microb. Ecol..

[9] Bellec L., Grimsley N., Derelle E., Moreau H., Desdevises Y. (2010). Abundance, spatial distribution and genetic diversity of *Ostreococcus tauri* viruses in two different environments. Environ. Microbiol. Rep..

[10] Bratbak G., Egge J.K., Heldal M. (1993). Viral mortality of the marine alga *Emiliania huxleyi* (Haptophyceae) and termination of algal blooms. Mar. Ecol. Prog. Ser..

[11] Bratbak G., Jacobsen A., Heldal M., Nagasaki K., Thingstad F. (1998). Virus production in *Phaeocystis pouchetii* and its relation to host cell growth and nutrition. Aquat. Microb. Ecol..

[12] Wilson W.H., Carr N.G., Mann N.H. (1996). The effect of phosphate status on the kinetics of cyanophage infection in the oceanic cyanobacterium *Synechococcus* sp. WH7803. J. Phycol..

[13] Maat D., Crawfurd K., Timmermans K., Brussard C. (2014). Elevated CO_2_ and phosphate limitation favor *Micromonas pusilla* through stimulated growth and reduced viral impact. Appl. Environ. Microbiol..

[14] Jacquet S., Bratbak G. (2003). Effects of ultraviolet radiation on marine virus-phytoplankton interactions. FEMS Microbiol. Ecol..

[15] Jacquet S., Heldal M., Iglesias-Rodriguez D., Larsen A., Wilson W., Bratbak G. (2002). Flow cytometric analysis of an *Emiliana huxleyi* bloom terminated by viral infection. Aquat. Microb. Ecol..

[16] Thyrhaug R., Larsen A., Brussaard C.P.D., Mcfadden P. (2002). Cell Cycle Dependent Virus Production in Marine Phytoplankton 1. Cell Cycle.

[17] Larsen J.B., Larsen A., Thyrhaug R., Bratbak G., Sandaa R.-A. (2007). Response of marine viral populations to a nutrient induced phytoplankton bloom at different pCO_2_ levels. Biogeosci. Discuss..

[18] Chen S., Gao K., Beardall J. (2014). Viral attack exacerbates the susceptibility of a bloom-forming alga to ocean acidification. Glob. Chang. Biol..

[19] Danovaro R., Corinaldesi C., Dell’Anno A., Fuhrman J.A., Middelburg J.J., Noble R.T., Suttle C.A. (2011). Marine viruses and global climate change. FEMS Microbiol. Rev..

[20] Bohannan B.J.M., Kerr B., Jessup C.M., Hughes J.B., Sandvik G. (2002). Trade-offs and coexistence in microbial microcosms. Antonie van Leeuwenhoek.

[21] Lenski R.E. (1988). Experimental Studies of Pleiotropy and Epistasis in *Escherichia coli.* I. Variation in Competitive Fitness Among Mutants Resistant to Virus T4. Evolution.

[22] Lennon J.T., Khatana S.A.M., Marston M.F., Martiny J.B.H. (2007). Is there a cost of virus resistance in marine cyanobacteria?. ISME J..

[23] Frickel J., Sieber M., Becks L. (2016). Eco-evolutionary dynamics in a coevolving host-virus system. Ecol. Lett..

[24] Seed K.D., Faruque S.M., Mekalanos J.J., Calderwood S.B., Qadri F., Camilli A. (2012). Phase Variable O Antigen Biosynthetic Genes Control Expression of the Major Protective Antigen and Bacteriophage Receptor in Vibrio cholerae O1. PLoS Pathog..

[25] León M., Bastías R. (2015). Virulence reduction in bacteriophage resistant bacteria. Front. Microbiol..

[26] Clerissi C., Desdevises Y., Grimsley N. (2012). Prasinoviruses of the marine green alga *Ostreococcus tauri* are mainly species specific. J. Virol..

[27] Marston M.F., Pierciey F.J., Shepard A., Gearin G., Qi J., Yandava C., Schuster S.C., Henn M.R., Martiny J.B.H. (2012). Rapid diversification of coevolving marine Synechococcus and a virus. Proc. Natl. Acad. Sci. USA.

[28] Avrani S., Wurtzel O., Sharon I., Sorek R., Lindell D. (2011). Genomic island variability facilitates *Prochlorococcus*-virus coexistence. Nature.

[29] Meyer J.R., Agrawal A.A., Quick R.T., Dobias D.T., Schneider D., Lenski R.E. (2010). Parallel changes in host resistance to viral infection during 45,000 generations of relaxed selection. Evolution.

[30] Avrani S., Lindell D. (2015). Convergent evolution toward an improved growth rate and a reduced resistance range in *Prochlorococcus* strains resistant to phage. Proc. Natl. Acad. Sci. USA.

[31] Avrani S., Schwartz D.A., Lindell D. (2012). Virus-host swinging party in the oceans: Incorporating biological complexity into paradigms of antagonistic coexistence. Mob. Genet. Elem..

[32] Field C.B. (1998). Primary Production of the Biosphere: Integrating Terrestrial and Oceanic Components. Science.

[33] Bellec L., Grimsley N., Moreau H., Desdevises Y. (2009). Phylogenetic analysis of new Prasinoviruses (*Phycodnaviridae*) that infect the green unicellular algae *Ostreococcus*, *Bathycoccus* and *Micromonas*. Environ. Microbiol. Rep..

[34] Derelle E., Ferraz C., Escande M.-L., Eychenié S., Cooke R., Piganeau G., Desdevises Y., Bellec L., Moreau H., Grimsley N. (2008). Life-cycle and genome of OtV5, a large DNA virus of the pelagic marine unicellular green alga *Ostreococcus tauri*. PLoS ONE.

[35] Thomas R., Grimsley N., Escande M.-L., Subirana L., Derelle E., Moreau H. (2011). Acquisition and maintenance of resistance to viruses in eukaryotic phytoplankton populations. Environ. Microbiol..

[36] Yau S., Hemon C., Derelle E., Moreau H., Piganeau G., Grimsley N. (2016). A Viral Immunity Chromosome in the Marine Picoeukaryote, *Ostreococcus tauri*. PLoS Pathog..

[37] Keller M.D., Selvin R.C., Claus W., Guillard R.R.L. (1987). Media for the culture of oceanic ultraphytoplankton. J. Phycol..

[38] Bates D., Mächler M., Bolker B., Walker S. (2014). Fitting Linear Mixed-Effects Models using lme4. J. Stat. Softw..

[39] Kuznetsova A., Brockhoff P.B., Christensen R.H.B. (2015). lmerTest: Tests in Linear Mixed Effects Models. http://CRAN.R-project.org/package=lmerTest.

[40] Schwarzländer M., Fricker M., Müller C., Marty L., Brach T., Novak J., Sweetlove L., Hell R., Meyer A. (2008). Confocal imaging of glutathione redox potential in living plant cells. J. Microsc..

[41] Corellou F., Schwartz C., Motta J.-P., Djouani-Tahri E.B., Sanchez F., Bouget F.-Y. (2009). Clocks in the green lineage: Comparative functional analysis of the circadian architecture of the picoeukaryote *Ostreococcus*. Plant Cell.

[42] Van Ooijen G., Knox K., Kis K., Bouget F.-Y., Millar A.J. (2012). Genomic Transformation of the Picoeukaryote *Ostreococcus tauri*. J. Vis. Exp..

[43] Heath S.E., Collins S. (2016). Mode of resistance to viral lysis affects host growth across multiple environments in the marine picoeukaryote *Ostreococcus tauri*. Environ. Microbiol..

[44] Schaum E., Collins S. (2014). Plasticity predicts evolution in a marine alga. Proc. R. Soc. B.

[45] Bell G. (2008). Selection: The Mechanism of Evolution.

[46] Lenski R.E. (1988). Experimental Studies of Pleiotropy and Epistasis in *Escherichia coli.* II. Compensation for Maldaptive Effects Associated with Resistance to Virus T4. Evolution.

[47] Björkman J., Nagaev I., Berg O.G., Hughes D., Andersson D.I. (2000). Effects of environment on compensatory mutations to ameliorate costs of antibiotic resistance. Science.

[48] Bellec L., Clerissi C., Edern R., Foulon E., Simon N., Grimsley N., Desdevises Y. (2014). Cophylogenetic interactions between marine viruses and eukaryotic picophytoplankton. BMC Evol. Biol..

[49] Luria S., Delbrück M. (1943). Mutations of Bacteria from Virus Sensitivity to Virus Resistance. Genetics.

[50] Karafistan A., Martin J.M., Rixen M., Beckers J.M. (2002). Space and time distributions of phosphate in the Mediterranean Sea. Deep. Sea Res. I.

[51] Jessup C.M., Bohannan B.J.M. (2008). The shape of an ecological trade-off varies with environment. Ecol. Lett..

[52] Bohannan B.J.M., Travisano M., Lenski R.E. (1999). Epistatic Interactions Can Lower the Cost of Resistance to Multiple Consumers. Evolution.

[53] Finkel Z.V., Beardall J., Flynn K.J., Quigg A., Rees T.A.V., Raven J.A. (2010). Phytoplankton in a changing world: Cell size and elemental stoichiometry. J. Plankton Res..

[54] Peter K.H., Sommer U. (2015). Interactive effect of warming, nitrogen and phosphorus limitation on phytoplankton cell size. Ecol. Evol..

[55] Atkinson D., Ciotti B.J., Montagnes D.J.S. (2003). Protists decrease in size linearly with temperature: Ca. 2.5% °C^−1^. Proc. Biol. Sci..

[56] Morán X.A.G., López-Urrutia Á., Calvo-Díaz A., Li W.K.W. (2010). Increasing importance of small phytoplankton in a warmer ocean. Glob. Chang. Biol..

[57] Geider R., Platt T., Raven J. (1986). Size dependence of growth and photosynthesis in diatoms: A synthesis. Mar. Ecol. Ser..

[58] Šupraha L., Gerecht A.C., Probert I., Henderiks J. (2015). Eco-physiological adaptation shapes the response of calcifying algae to nutrient limitation. Sci. Rep..

[59] Ryther J., Menzel D. (1959). Light adaptation by marine phytoplankton. Limnol. Oceanogr..

[60] Wozniak B., Hapter R., Dera J. (1989). Light curves of marine plankton photosynthesis in the Baltic. Oceanologia.

[61] Renk H., Ochocki S. (1998). Photosynthetic rate and light curves of phytoplankton in the southern Baltic. Oceanologia.

[62] Riemann B., Simonsen P., Stensgaard L. (1989). The carbon and chlorophyll content of phytoplankton from various nutrient regimes. J. Plankton Res..

[63] McLachlan J. (1961). The effect of salinity on growth and chlorophyll content in representative classes of unicellular marine algae. Can. J. Microbiol..

[64] Sigaud T.C.S., Aidar E. (1993). Salinity and temperature effects on the growth and chlorophyll-a content of some planktonic aigae. Bol. Inst. Oceanogr..

[65] Brockhurst M.A., Rainey P.B., Buckling A. (2004). The effect of spatial heterogeneity and parasites on the evolution of host diversity. Proc. Biol. Sci..

