# Supplementary material for: Virus Resistance Is Not Costly in a Marine Alga Evolving under Multiple Environmental Stressors
Source: Viruses. 2017 Mar 8;9(3):39. doi: 10.3390/v9030039 (PMC5371794; doi:10.3390/v9030039)
Supplement: Supplementary file 1 [file viruses-09-00039-s001.docx]

**Supplementary Materials: Virus Resistance Is Not Costly in a Marine Alga Evolving under Multiple Environmental Stressors**

**Sarah E. Heath, Kirsten Knox, Pedro F. Vale and Sinead Collins**


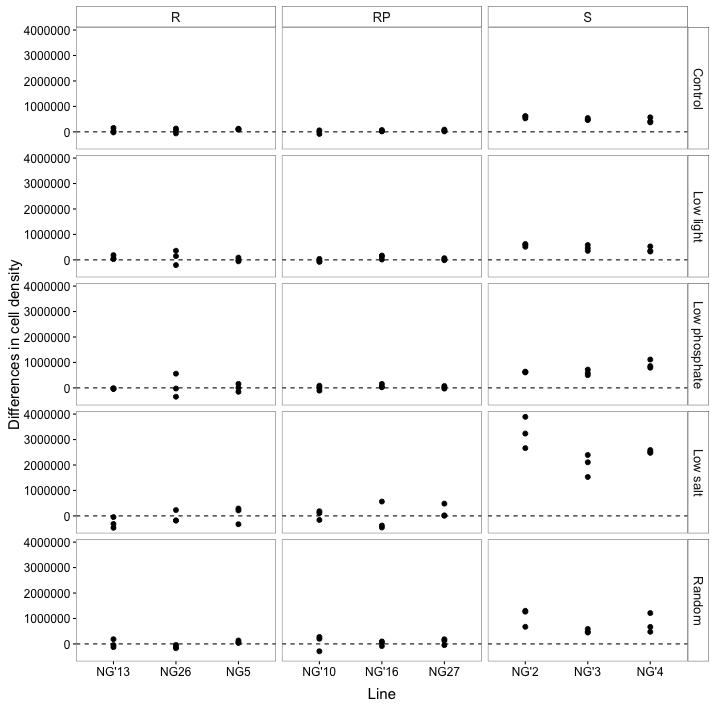


**Figure S1**. Differences in cell densities between the populations that were not inoculated with OtV5 and populations that were inoculated. The dashed line represents no difference.


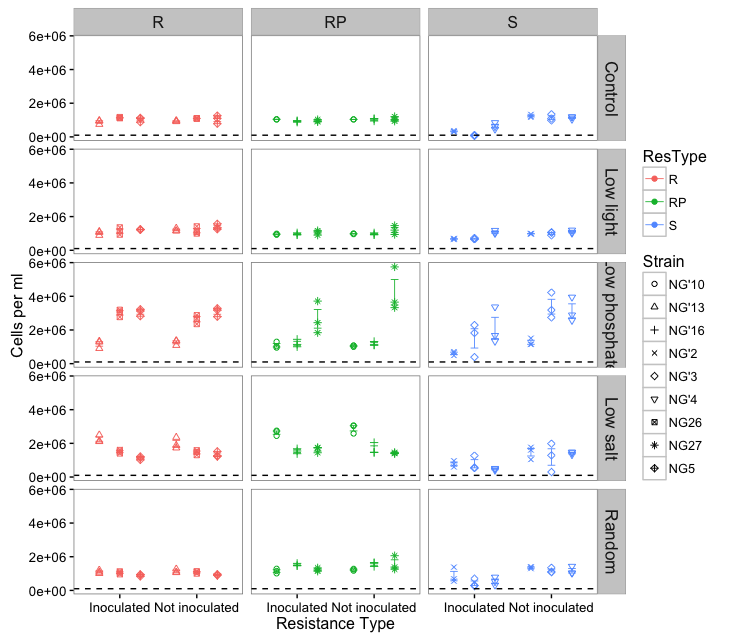


**Figure S2**. Mean (± SE) cell density ml^-1^ of resistant (R), resistant producer (RP) and susceptible (S) O. tauri lines 7 days after OtV5 inoculation in five environments. Points represents the average of the three assay replicates for each evolved population. Inoculated = popuations inoculated with OtV5, Not inoculated = negative control populations that were grown for the same period without OtV5 inoculation. There were three evolved populations of each line. The dashed line represents the starting cell density at 100 000 cell ml^-1^.


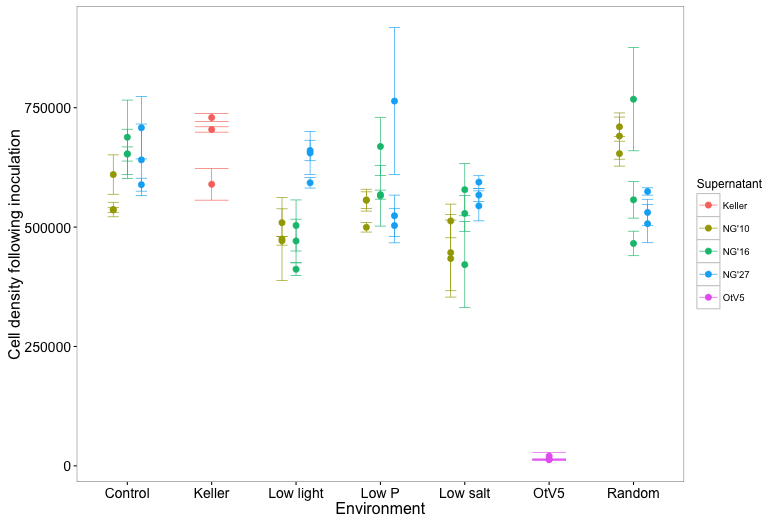


**Figure S3**. Cell density of *O. tauri* strain RCC4221 after inoculation with supernatant from three resistant producer lines NG’10, NG’16 and NG27. Inoculation with Keller media was used as a negative control and OtV5 was used as a positive control.


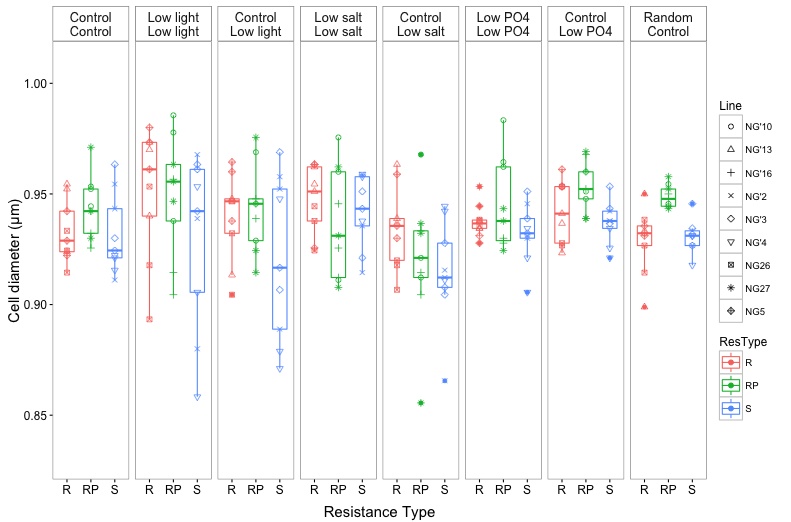


**Figure S4**. Mean cell size of O. tauri populations evolved and assayed under different environments. R = resistant, RP= resistant producer, S= susceptible. Each panel represents an assay, with populations evolved in the selection environment (top label) and cell size measured in the assay environment (bottom label).


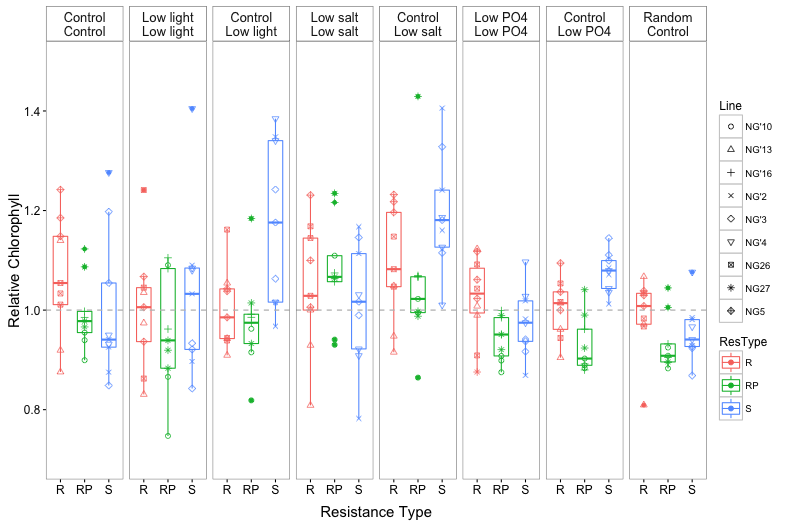


**Figure S5**. Relative chlorophyll content per cell volume of O. tauri populations evolved and assayed under different environments. R = resistant, RP= resistant producer, S= susceptible. Each panel represents an assay, with populations evolved in the selection environment (top label) and mean chlorphyll content per cell measured in the assay environment (bottom label).

© 2017 by the authors. Licensee MDPI, Basel, Switzerland. This article is an open access article distributed under the terms and conditions of the Creative Commons by Attribution (CC-BY) license (http://creativecommons.org/licenses/by/4.0/).
